# Efficacy of a Standardized Ginger Extract for Menstrual Pain and Associated Symptoms in Women With Primary Dysmenorrhea: A Randomized, Double-Blind, Placebo-Controlled Trial

**DOI:** 10.7759/cureus.114903

**Published:** 2026-08-21

**Authors:** Prashanth Kumar Babu, Abhimanyu Prashar, Pushank Nagar, K Hemalatha, Himanshu Trikambhai Makwana, Rakesh Kumar Yadav, Jayant Deshpande, Mohammed Shuaib Khan, Nehru Sai Suresh Chalichem

**Affiliations:** 1 Research and Development, Ennature Biopharma, India Glycols Limited, Dehradun, IND; 2 Obstetrics and Gynecology, Udhbhava Hospital, Bangalore, IND; 3 Research and Development, Edison Life Sciences LLC, Piscataway, USA

**Keywords:** ginger extract, menstrual fluid flow, menstrual pain, primary dysmenorrhea, prostaglandin f2α

## Abstract

Background: Primary dysmenorrhea, a common gynecological condition characterized by painful menstrual cramps, significantly impairs quality of life. Ginger has traditionally been used for pain relief and is proposed to exert anti-inflammatory and analgesic effects. This study aimed to evaluate the efficacy and safety of a standardized ginger extract compared with placebo in women with primary dysmenorrhea.

Methods: In this randomized, double-blind, placebo-controlled, multicenter clinical study, participants with primary dysmenorrhea received either a standardized ginger extract or placebo twice daily for 90 days. The primary endpoints were changes in pain severity, assessed using the Visual Analog Scale (VAS), and dysmenorrhea severity, assessed using the Working Ability, Location, Intensity, Days of Pain, Dysmenorrhea (WaLIDD) score. Secondary endpoints included changes in menstrual symptom burden, assessed using the Menstrual Distress Questionnaire (MEDI-Q), menstrual fluid flow, and global assessments. Exploratory endpoints included prostaglandin F2α (PGF2α) levels. Efficacy analyses were performed in the intention-to-treat (ITT) and per-protocol (PP) populations.

Results: Compared with placebo (n = 47), standardized ginger extract (n = 49) significantly reduced pain severity and overall dysmenorrhea severity from Cycle 1 onward. At Cycle 3, VAS and WaLIDD scores were reduced by 31.2%-33.3% and 62.4%-63.5%, respectively, from baseline (p < 0.001). Significant improvements in menstrual symptom burden and menstrual fluid flow (31.9% reduction) were observed compared with placebo (p < 0.05 to p < 0.001). PGF2α levels decreased significantly by 11.6%-11.9% in the ginger extract group, whereas they increased in the placebo group (p < 0.001). The intervention was well tolerated, with no clinically significant adverse events reported.

Conclusion: Standardized ginger extract significantly reduced pain severity, dysmenorrhea severity, menstrual symptom burden, and menstrual fluid flow in women with primary dysmenorrhea. Its favorable safety and tolerability profile support its potential utility as a non-hormonal therapeutic approach for the management of primary dysmenorrhea.

## Introduction

Primary dysmenorrhea is one of the most common gynecological conditions affecting women of reproductive age. It is characterized by recurrent, cramping lower abdominal pain occurring in the absence of identifiable pelvic pathology. The condition is often accompanied by a range of systemic and psychological symptoms, including low back pain, fatigue, nausea, vomiting, headache, and mood disturbances [1,2]. The global prevalence of primary dysmenorrhea is high, with estimates suggesting that approximately 73% of menstruating women experience symptoms. Across continents, the highest prevalence has been reported in Central America and Oceania, at 89.6% and 75.8%, respectively, whereas North America has been reported to have the lowest prevalence, at 59.4% [3]. Primary dysmenorrhea can significantly affect daily activities, work productivity, and overall quality of life [1]. Its pathophysiology is primarily attributed to increased synthesis and release of uterine prostaglandins, particularly prostaglandin F2α (PGF2α), resulting in increased uterine contractility and ischemic pain [4]. Accordingly, treatment strategies have traditionally focused on reducing prostaglandin production or modulating uterine activity.

Current management options include nonsteroidal anti-inflammatory drugs (NSAIDs) and hormonal therapies, such as oral contraceptives. Although NSAIDs are widely used and effective in many cases, their use is associated with neurological symptoms (e.g., headache, drowsiness, and dizziness), gastrointestinal symptoms (e.g., nausea and indigestion), and hepatic, renal, and cardiovascular effects, particularly with long-term use. Approximately 20% of patients with dysmenorrhea have been reported to be unresponsive to NSAID treatment, a condition referred to as NSAID-resistant dysmenorrhea [1,2,5]. Hormonal therapies, although effective, may not be suitable for all individuals because of contraindications, adverse effects, or personal preferences, including concerns regarding hormonal exposure. Additionally, these treatments may not adequately address the broader spectrum of symptoms associated with dysmenorrhea, including psychological and quality-of-life impairments [6].

In this context, there is increasing interest in herbal or plant-based interventions as alternative or complementary approaches to the management of dysmenorrhea [7]. Such interventions are often perceived as safer [5] and may exert multiple effects, including anti-inflammatory, analgesic, and antioxidant activities [7,8]. Ginger (*Zingiber officinale*) is a widely used medicinal plant with a long history of use in traditional medicine for the management of pain, inflammation, and gastrointestinal disorders [9]. Bioactive constituents of ginger, including gingerols and shogaols, have been shown to inhibit cyclooxygenase and lipoxygenase pathways, thereby reducing prostaglandin synthesis [10]. Preclinical and clinical studies have suggested that ginger may be effective in alleviating menstrual pain [9-11]. However, many existing studies are limited by short treatment durations, small sample sizes, or a primary focus on pain outcomes without evaluating broader clinical, functional, and mechanistic parameters. Furthermore, evidence regarding the effects of ginger on biological markers such as PGF2α remains limited, despite their potential relevance to understanding its mechanism of action and safety.

Therefore, the present study was designed to comprehensively evaluate the efficacy and safety of a standardized ginger extract in women with primary dysmenorrhea using a multidimensional approach encompassing clinical, patient-reported, and mechanistic outcomes. The primary objective of this study was to evaluate the safety and efficacy of ginger extract granules compared with placebo in participants with primary dysmenorrhea. The secondary objectives were to evaluate changes in physical, mood, and behavioral symptoms, including the intensity of menstrual cramps, low back pain, fatigue, nausea and vomiting, lack of energy, mood swings, and headache; menstrual fluid flow; the incidence of adverse events; and participants' and physicians' global assessment scores.

## Materials and methods

Trial design and participants

This randomized, double-blind, placebo-controlled, multicenter clinical trial was conducted from May 2025 to September 2025 to evaluate the efficacy and safety of a standardized ginger extract in otherwise healthy women with primary dysmenorrhea. The study protocol, informed consent form, and all associated documents were approved by the Pranav Diabetes Center Ethics Committee (PDCEC/SRS- CTSRS/ 2501-01/17 Apr 25) prior to study initiation. The trial was conducted in accordance with the International Council for Harmonisation (ICH) Good Clinical Practice E6 (R3), applicable regulatory guidelines, the ethical principles of the 2024 Declaration of Helsinki, and the New Drugs and Clinical Trials Rules, 2019. The trial was prospectively registered with the Clinical Trials Registry-India (CTRI/2025/05/086338). All participants provided written informed consent before undergoing any study-related procedures.

Eligible participants were healthy, married women aged 20-30 years who were sexually active, had no history of childbirth or abortion, and resided in metropolitan areas. Participants were required to have a history of primary dysmenorrhea for at least two consecutive menstrual cycles prior to screening, with moderate pain intensity, defined as a Visual Analog Scale (VAS) score of ≥4 to ≤6, during at least four of the previous six menstrual cycles. Participants were required to have no known allergy to the investigational product, be willing to use menstrual cups throughout the study, and agree to use condoms as the sole contraceptive method. Key exclusion criteria included secondary dysmenorrhea, fertility-related disorders, a history of severe gastrointestinal conditions (e.g., gastritis, gastrointestinal bleeding, or ulceration), urinary tract infection, a positive pregnancy test, breastfeeding, a history of abortion, and hypoactive sexual desire disorder.

The study spanned five menstrual cycles, including two pre-dosing cycles (C-1 and C-2) for baseline assessment and three post-dosing cycles (C1, C2, and C3) following randomization and initiation of treatment. A total of 11 study visits were scheduled, comprising two visits per menstrual cycle (day 1 and day 3) and one enrollment and randomization visit (day 5 of cycle C-2). Baseline evaluations of all dysmenorrhea-related outcomes were conducted during the pre-dosing cycles.

Interventions

The investigational product and matching placebo capsules were manufactured by Ennature Biopharma, a division of India Glycols Limited, Dehradun, India. Each investigational product capsule contained 100 mg of ginger extract granules with food-grade excipients and was standardized to ≥20% (w/w) total gingerols, as determined by high-performance liquid chromatography (HPLC). The extract was derived from the dried rhizomes of *Zingiber officinale*. Participants assigned to the placebo group received capsules containing microcrystalline cellulose, which were matched to the investigational product in size, shape, and color. Participants were instructed to take the assigned intervention orally twice daily after meals for 90 consecutive days. Treatment compliance was assessed by counting returned capsules at each study visit.

Sample size and randomization

The sample size was calculated based on a previously published study in participants with primary dysmenorrhea [12], which reported mean (SD) pain scores of 5.12 (2.69) for the ginger group and 6.58 (2.02) for the placebo group. A total sample size of 100 participants (50 per group) was planned to achieve 80% power at a two-sided alpha level of 0.05, assuming a 20% dropout rate. Each participant was assigned a unique identification number. Randomization was performed in a 1:1 ratio using a block randomization method and a web-based randomization tool (GraphPad Prism version 10.6.1; GraphPad Software, San Diego, CA, USA). Sealed, participant-specific envelopes containing the randomization codes were used for treatment allocation. The investigational product and placebo were identical in appearance to maintain blinding, and both investigators and participants were blinded to treatment allocation.

Dysmenorrhea-related outcome measures

Primary Outcomes

Pain severity by VAS: The primary endpoint was the change in dysmenorrhea pain severity, assessed using the VAS, a validated unidimensional measure of pain intensity. The VAS consists of a 10-cm horizontal line anchored at “no pain” (score = 0) and “worst imaginable pain” (score = 10). Participants marked a point on the line corresponding to their menstrual pain intensity, and the distance from the “no pain” anchor was measured in centimeters. Higher scores indicated greater pain intensity. Assessments were performed at each study visit, except the randomization visit [13,14].

Dysmenorrhea severity by Working Ability, Location, Intensity, Days of Pain, Dysmenorrhea (WaLIDD) score: The severity of dysmenorrhea was further evaluated using the WaLIDD score, a multidimensional tool designed to assess the overall burden of menstrual pain [15,16]. The WaLIDD score comprises four domains: working ability, pain location, pain intensity, and the number of days with pain. Each domain is scored on an ordinal scale from 0 to 3, and the individual domain scores are summed to generate a total score ranging from 0 to 12, with higher scores indicating greater dysmenorrhea severity. Assessments were performed at each study visit. Both domain-specific scores and total WaLIDD scores were analyzed to evaluate changes in the multidimensional severity and functional impact of dysmenorrhea.

Secondary Outcomes

Menstrual symptom burden by the Menstrual Distress Questionnaire (MEDI-Q): Menstrual symptom burden was assessed using the MEDI-Q, a structured, patient-reported outcome measure designed to capture the severity and impact of menstrual-related symptoms across physical, emotional, and behavioral domains [17,18]. The instrument comprises subscales assessing menstrual symptoms, menstrual symptom distress, and the menstrual specificity index. Each item is rated on a graded scale reflecting symptom severity, and the item scores are summed to generate a total score, with higher scores indicating a greater symptom burden. Assessments were performed at each study visit to evaluate changes in overall menstrual symptom burden across menstrual cycles.

Menstrual fluid flow: Menstrual fluid flow was assessed using a semi-quantitative approach based on menstrual cup use. Participants were instructed to use menstrual cups throughout the study and record the volume of menstrual fluid collected during each cycle. Menstrual fluid volume was measured using the calibrated markings on the menstrual cup and recorded at specified time points during menstruation. Changes in menstrual fluid volume from baseline were analyzed to assess the effect of the intervention on menstrual bleeding patterns.

Global assessments: Both participant- and physician-reported global assessments were performed to capture the overall treatment effects on dysmenorrhea-related symptoms. The Subject's Global Assessment of Symptoms evaluated the participant's overall perception of improvement in dysmenorrhea-related symptoms using an ordinal scale, with higher scores indicating greater perceived improvement. The Physician's Global Assessment of Response to Supplementation evaluated the investigator's overall assessment of the participant's response to the intervention, while the Physician's Global Impression of Change assessed the overall change in the participant's clinical condition relative to baseline. All global assessments were performed during each post-baseline menstrual cycle (C1, C2, and C3).

**Figure 1:**
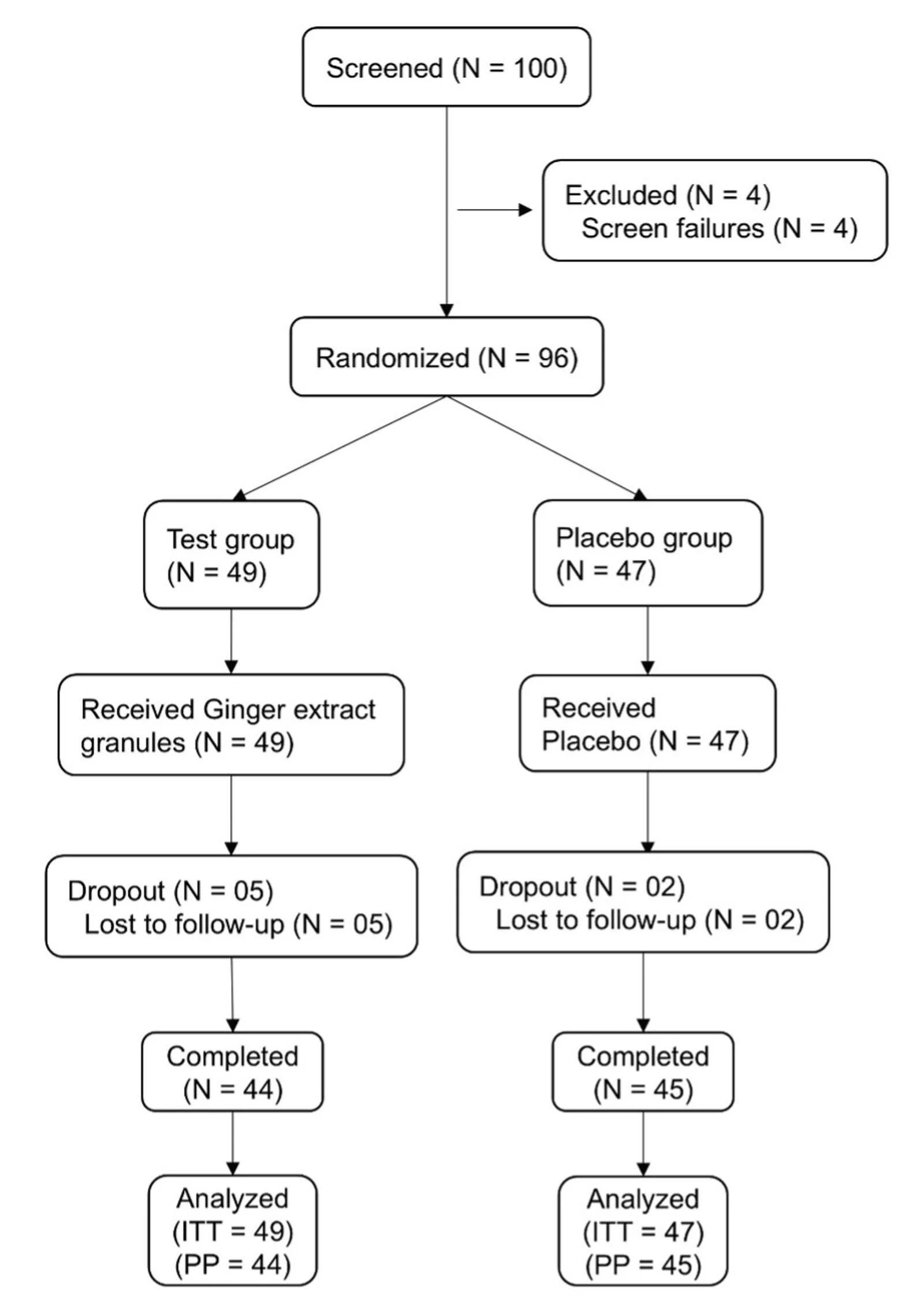
Flow of participants in the study and allocation of intervention ITT: intention-to-treat, PP: per-protocol.

Biomarker assessment: Exploratory endpoints included changes in PGF2α and estradiol levels. PGF2α assessments were conducted on day 1 of each cycle, except during the final post-dosing cycle. Estradiol levels were assessed on day 3 of each cycle. These biomarkers were evaluated to provide mechanistic insights into the potential effects of the intervention on the pathophysiology of dysmenorrhea and to assess the potential for endocrine disruption. The study methodology is presented in Figure 1.

Safety monitoring

Adverse events were monitored throughout the study and recorded in the Case Report Form from the time of informed consent through study completion. Adverse events were coded using the Medical Dictionary for Regulatory Activities (MedDRA) and graded for severity according to the Common Terminology Criteria for Adverse Events (CTCAE), version 5.0. Vital signs, including body temperature, blood pressure, pulse rate, and respiratory rate, were assessed at all study visits. Safety laboratory evaluations, including hematological parameters, liver function tests, renal function tests, and urinalysis, were performed during the pre-dosing period and at the end of the study.

Statistical analysis

All data were entered into Microsoft Excel (Microsoft Corp., Redmond, WA, USA) and analyzed using IBM SPSS Statistics for Windows, Version 26.0 (Released 2018; IBM Corp., Armonk, NY, USA). Descriptive and inferential statistics were performed for continuous and ordinal variables, as appropriate. Between-group comparisons were performed using the independent-samples t-test or Mann-Whitney U test, depending on the distribution of the data. Statistical significance was defined as a two-sided alpha level of 0.05. Missing data were handled using the last observation carried forward (LOCF) method. Participants were categorized into intention-to-treat (ITT), per-protocol (PP), and safety populations. The ITT population included all randomized participants. The PP population included participants who completed the study according to the protocol and met the predefined protocol-compliance criteria. The safety population included all participants who received at least one dose of the study intervention.

## Results

A total of 96 participants were included in the ITT analysis, comprising 49 (51.0%) participants in the test group and 47 (49.0%) in the placebo group. Baseline demographic characteristics were well balanced between groups (Table 1), with no statistically significant differences observed for age, body weight, height, or body mass index (BMI) (all p > 0.05). The mean age was 25.08 ± 2.62 years in the test group and 25.00 ± 2.34 years in the placebo group. Similarly, mean body weight was 60.12 ± 3.25 kg and 61.12 ± 4.00 kg, while mean BMI was 24.31 ± 1.92 kg/m² and 24.72 ± 2.22 kg/m², respectively. None of the enrolled participants reported significant medical history or concomitant medication use during the study, indicating a clinically comparable study population before intervention.

**Table 1:** Baseline characteristics of the intention-to-treat (ITT) population Values are presented as mean ± SD.

Characteristics	Test (n = 49; 51.0%)	Placebo (n = 47; 49.0%)
Age (years)	25.08 ± 2.62	25.00 ± 2.34
Body weight (kg)	60.12 ± 3.25	61.12 ± 4.00
Height (cm)	157.47 ± 4.95	157.49 ± 6.35
BMI (kg/m²)	24.31 ± 1.92	24.72 ± 2.22

Pain severity (VAS)

Baseline VAS scores were comparable between the test (5.36 ± 0.56) and placebo (5.30 ± 0.58) groups. Following treatment, the test group demonstrated a progressive and highly significant reduction in pain severity, with percentage reductions of 12.5%, 13.3%, and 31.2% at cycles C1, C2, and C3, respectively (p < 0.001 for all). In contrast, the placebo group showed only minimal improvement, with a percentage reduction of 2.5% at C1, followed by 6.8% at C2, while pain scores increased by 6.0% at C3. Between-group comparisons showed significantly greater pain reduction in the test group at every post-treatment cycle, resulting in a placebo-adjusted improvement of approximately 37.2 percentage points at the end of treatment. Similar findings were observed in the PP analysis.

Dysmenorrhea severity (WaLIDD score)

The baseline WaLIDD total score was similar between groups (test: 5.77 ± 0.72; placebo: 5.80 ± 0.79). Treatment produced marked improvements in the test group, with percentage reductions of 44.2%, 44.2%, and 62.4% at C1, C2, and C3 (p < 0.001 throughout). Conversely, placebo recipients experienced no meaningful improvement, with slight worsening during follow-up. Consequently, the placebo-adjusted difference reached approximately 79.5 percentage points at the final cycle. Analysis of individual WaLIDD domains demonstrated consistent superiority of the test intervention. Working ability improved by 45.6%, 49.3%, and 68.4%; pain location by 45.0%, 43.6%, and 61.1%; pain intensity by 42.5%, 42.5%, and 63.3%; and duration of pain by 43.1%, 41.2%, and 57.5% across C1-C3, respectively (all p < 0.001). Placebo participants showed either negligible improvement or worsening of these domains over time. These findings indicate that the intervention substantially reduced the severity, distribution, intensity, and duration of dysmenorrhea while simultaneously improving participants' functional ability. These parameters are presented in Table 2. 

**Table 2:** Changes in outcome scores across groups in the intention-to-treat population (n = 96) Values are presented as mean ± SD. ^*^Statistical significance was defined as a two-sided p-value < 0.05. ^†^Between-group comparisons were performed using an independent-samples t-test. VAS: Visual Analog Scale, WaLIDD: Working Ability, Location, Intensity, Days of Pain, Dysmenorrhea.

Outcome	Baseline test (n = 49)	Baseline placebo (n = 47)	t‑statistic^†^	p-value^*^
VAS pain	5.36 ± 0.56	5.30 ± 0.58	5.15	<0.001
WaLIDD total	5.77 ± 0.72	5.80 ± 0.79	-19.4	<0.001
Working ability	1.36 ± 0.32	1.46 ± 0.37	-14.14	<0.001
Pain location	1.49 ± 0.34	1.38 ± 0.29	17.08	<0.001
Pain intensity	1.39 ± 0.33	1.40 ± 0.28	16.0	<0.001
Days of pain	1.53 ± 0.34	1.57 ± 0.35	-5.67	<0.001

Menstrual symptom burden (MEDI-Q)

Baseline MEDI-Q scores were comparable between study groups. The test group demonstrated a rapid and sustained reduction in menstrual symptom burden, with percentage reductions of 93.8%, 92.9%, and 95.3% in total MEDI-Q score at C1, C2, and C3, respectively (p < 0.001). Although the placebo group also improved over time (30.8%, 84.1%, and 89.2% reductions), symptom resolution occurred considerably later, and between-group differences remained statistically significant throughout follow-up.

Similarly, menstrual symptom scores declined by 89.2%, 80.2%, and 87.4% in the test group compared with 24.7%, 70.4%, and 80.3% in the placebo group. Menstrual symptom distress also improved substantially, with percentage reductions of 53.0%, 67.5%, and 75.8% in the intervention arm versus 14.0%, 49.7%, and 52.3% in the placebo arm. Likewise, menstrual specificity index decreased by 43.7%, 72.4%, and 64.4% in the test group, compared with 9.0%, 53.9%, and 62.9% in the placebo group. While both groups achieved similar menstrual specificity scores by the final cycle, the intervention produced a significantly faster onset of clinical benefit. The menstrual outcomes are provided in Table 3.

**Table 3:** Changes in Menstrual Distress Questionnaire (MEDI-Q) scores across groups in the intention-to-treat population Values are presented as mean ± SD. ^*^Statistical significance was defined as a two-sided p-value < 0.05. ^†^Between-group comparisons were performed using an independent-samples t-test.

Cycles	Test group (n = 49)	Placebo group (n = 47)	t‑statistic^†^	p‑value^*^
MEDI‑Q total score				
Baseline	46.50 ± 5.19	47.45 ± 4.63	-0.95	0.346
C1	2.90 ± 2.48	32.84 ± 14.63	-13.84	<0.001
C2	3.30 ± 2.30	7.53 ± 4.60	-5.66	<0.001
C3	2.21 ± 2.36	5.13 ± 4.20	-4.18	<0.001
MEDI‑Q menstrual symptoms				
Baseline	15.53 ± 1.61	15.50 ± 1.40	0.10	0.923
C1	1.68 ± 1.42	11.68 ± 4.24	-15.38	<0.001
C2	3.08 ± 2.27	4.59 ± 2.35	-3.21	0.002
C3	1.96 ± 2.12	3.06 ± 2.42	-2.36	0.020
MEDI‑Q menstrual symptoms distress				
Baseline	3.02 ± 0.30	3.08 ± 0.26	-1.05	0.296
C1	1.42 ± 0.98	2.65 ± 0.70	-7.10	<0.001
C2	0.98 ± 0.39	1.55 ± 0.34	-7.65	<0.001
C3	0.73 ± 0.59	1.47 ± 0.82	-5.07	<0.001
MEDI‑Q menstrual specificity index				
Baseline	0.87 ± 0.16	0.89 ± 0.11	-0.72	0.476
C1	0.49 ± 0.42	0.81 ± 0.18	-4.88	<0.001
C2	0.24 ± 0.42	0.41 ± 0.31	-2.26	0.026
C3	0.31 ± 0.42	0.33 ± 0.38	-0.25	0.807

Menstrual fluid flow

In the ITT population, baseline menstrual fluid flow was comparable between the test (51.14 ± 13.62) and placebo (52.90 ± 9.56) groups (Table 4). The test group showed consistent reductions across post-dosing cycles, corresponding to a reduction of 19.6% at C1, 26.3% at C2, and 31.9% at C3 from baseline (p < 0.01 at C1 and p < 0.001 at C2 and C3). The placebo group demonstrated a progressive increase in menstrual fluid flow, corresponding to an increase of 8.1% at C1 (p < 0.05), 12.6% at C2 (p < 0.001), and 17.1% at C3 (p < 0.001). Between-group comparisons using independent samples t‑test confirmed significantly greater reductions in the test group compared with placebo at all time points (p < 0.0001 throughout) (Table 4).

**Table 4:** Menstrual fluid flow across groups in the intention-to-treat population (n = 96) Values are presented as mean ± SD. ^*^Statistical significance was defined as a two-sided p-value < 0.05. ^†^Between-group comparisons were performed using an independent-samples t-test.

Cycles	Test group (n = 49)	Placebo group (n = 47)	t‑statistic^†^	p‑value^*^
Baseline	51.14 ± 13.62	52.90 ± 9.56	-0.74	0.463
C1	41.12 ± 10.59	57.19 ± 9.90	-7.73	<0.001
C2	37.69 ± 9.33	59.55 ± 10.36	-10.88	<0.001
C3	34.82 ± 7.84	61.96 ± 9.83	-14.79	<0.001

Subject's Global Assessment of Symptoms

The test group demonstrated higher assessment scores than placebo at all time points (C1: 3.90 ± 0.68 vs 2.47 ± 0.72; C2: 4.00 ± 0.65 vs 2.98 ± 0.71; C3: 4.45 ± 0.61 vs 2.11 ± 0.94), with statistically significant between-group differences at all cycles (p < 0.001 throughout) in the ITT population.

Physician's Global Assessment of Response to Supplementation

The test group showed significantly higher response scores at C1 (2.67 ± 0.52 vs 2.00 ± 0.69; p < 0.0001) and C3 (3.45 ± 0.58 vs 1.83 ± 0.73; p < 0.001), while no significant difference was observed at C2 (2.94 ± 0.69 vs 2.64 ± 0.99; p = 0.104) in the ITT population (Table 5). 

**Table 5:** Global assessments by subject and physician across groups in the intention-to-treat population (n = 96) Values are presented as mean ± SD. ^*^Statistical significance was defined as a two-sided p-value < 0.05. ^†^Between-group comparisons were performed using an independent-samples t-test.

Cycles	Test group (n = 49)	Placebo group (n = 47)	t‑statistic^†^	p‑value^*^
Subject‑reported global assessment scores				
C1	3.90 ± 0.68	2.47 ± 0.72	9.98	<0.001
C2	4.00 ± 0.65	2.98 ± 0.71	7.30	<0.001
C3	4.45 ± 0.61	2.11 ± 0.94	14.37	<0.001
Physician's Global Assessment - response to supplementation				
C1	2.67 ± 0.52	2.00 ± 0.69	5.33	<0.001
C2	2.94 ± 0.69	2.64 ± 0.99	1.64	0.104
C3	3.45 ± 0.58	1.83 ± 0.73	12.15	<0.001
Physician's Global Assessment - global impression of change				
C1	2.47 ± 0.50	3.55 ± 0.50	-10.55	<0.001
C2	2.53 ± 0.50	3.45 ± 0.88	-6.28	<0.001
C3	2.29 ± 0.58	3.77 ± 0.94	-9.22	<0.001

PGF2α levels

Baseline levels of PGF2α in the ITT population were comparable between the test (216.68 ± 26.05 pg/mL) and placebo (216.18 ± 23.14 pg/mL) groups. The test group showed consistent reductions (3.7% at C1, 8.6% at C2, and 11.6% at C3), with statistical significance at C1, C2 and C3. The placebo group demonstrated progressive increases (2.6% at C1, 4.3% at C2, and 7.0% at C3), with significant changes at C2 and C3. Between-group comparisons using independent samples t‑test confirmed significantly lower PGF2α levels in the test group at all time points (C1: p < 0.01; C2 and C3: p < 0.001). 

Estradiol levels

Baseline estradiol levels in the ITT population were comparable between the test (105.64 ± 31.08 pg/mL) and placebo (91.86 ± 29.20 pg/mL) groups. The test group showed minimal variation across cycles, with estradiol levels remaining stable and not significantly different from baseline. The placebo group also demonstrated no meaningful changes across cycles. Between-group comparison using independent samples t‑test showed a statistically significant difference at baseline (p = 0.028), but no significant differences were observed at C1, C2, or C3. Overall, estradiol levels remained stable in both groups, indicating no treatment-related effect on circulating estradiol (Table 6).

**Table 6:** Prostaglandin F2α and estradiol levels across groups in the intention-to-treat population (n = 96) Values are presented as mean ± SD. ^*^Statistical significance was defined as a two-sided p-value < 0.05. ^†^Between-group comparisons were performed using an independent-samples t-test.

Cycles	Test group (pg/mL, n = 49)	Placebo group (pg/mL, n = 47)	t‑statistic^†^	p‑value^*^
PGF2α levels				
Baseline	216.68 ± 26.05	216.18 ± 23.14	0.10	0.921
C1	208.63 ± 23.73	221.85 ± 21.49	-2.86	0.005
C2	198.12 ± 22.26	225.55 ± 20.97	-6.17	<0.001
C3	191.53 ± 21.97	231.23 ± 20.66	-9.09	<0.001
Estradiol levels				
Baseline	105.64 ± 31.08	91.86 ± 29.20	2.23	0.028
C1	105.67 ± 29.16	95.06 ± 29.16	1.77	0.080
C2	105.39 ± 28.25	95.15 ± 28.65	1.77	0.080
C3	103.67 ± 26.30	94.74 ± 27.69	1.62	0.109

A total of seven subjects (14.29%) in the test group and five subjects (10.64%) in the placebo group reported adverse events during the study. There was no statistically significant difference in the overall incidence of adverse events between the groups (χ² = 0.182, df = 1, p = 0.75). All adverse events were mild in severity, transient in nature, and resolved without any medical intervention. The types of adverse events reported were consistent with common self‑limiting illnesses and showed no apparent pattern suggestive of treatment‑related toxicity. No adverse events resulted in dose modification, interruption, or discontinuation of the investigational product. No serious adverse events were reported during the study. Overall, the test substance was well tolerated, with no treatment‑related safety concerns (Table 7).

**Table 7:** Comparison of adverse events across groups in the intention-to-treat population (n = 96) Statistical significance was defined as a two-sided p-value < 0.05. Between-group comparisons were performed using the chi-square test or Fisher's exact test, as appropriate.

Adverse events	Test (n = 49)	Placebo (n = 47)	Chi-square value	p-value
Abdominal discomfort	0	1	0.182	0.75
Cold, cough, and fever	1	0		
Cold and fever	1	1		
Dry throat	0	1		
Fever	1	0		
Fever and dry mouth	1	0		
Fever and dry throat	1	0		
Headache	1	1		
Nausea	0	1		
Runny nose	1	0		
Total n (%)	7 (14.29%)	5 (10.64%)		

## Discussion

The present randomized, double-blind, placebo-controlled clinical study demonstrated that a standardized ginger extract significantly improved multiple dysmenorrhea-related outcomes compared with placebo in women with primary dysmenorrhea. The intervention resulted in clinically meaningful reductions in pain severity, overall dysmenorrhea severity, menstrual symptom burden, and menstrual fluid flow, while also providing mechanistic insights through reductions in PGF2α levels. These benefits were sustained over three consecutive menstrual cycles, highlighting the durability of the treatment effect.

The primary endpoint, reduction in pain severity as assessed using the VAS, showed a consistent and statistically significant decrease in the ginger group from Cycle 1 onward, with a placebo-adjusted difference of 37.2 percentage points at the end of the study. At Cycle 3, the test group demonstrated a 31.2% reduction in VAS scores from baseline, whereas the placebo group showed a 6.0% increase, indicating a clear divergence between the two groups. Although no firmly established minimal clinically important difference (MCID) for VAS scores in women with primary dysmenorrhea has been established, the magnitude of the reduction observed in the present study, in conjunction with the baseline VAS values, suggests that the improvement may be clinically meaningful from Cycle 1 onward [19]. Several clinical trials and systematic reviews have reported that ginger supplementation significantly reduces menstrual pain in women with primary dysmenorrhea [9,12,20]. Specifically, the reduction in pain severity assessed using the VAS in the present study was consistent with findings from previously published studies [21,22]. However, many earlier studies were limited by short intervention durations, the absence of placebo control, single-blind designs, or the use of insufficiently characterized ginger preparations [21,22]. In contrast to prior studies that primarily evaluated short-term pain outcomes over one or two menstrual cycles, the present study demonstrated sustained improvements across multiple menstrual cycles using a rigorously characterized standardized ginger extract.

The reduction in pain severity was further supported by improvements in WaLIDD scores, including reductions in pain intensity, duration, and functional impairment related to working ability. In the test group, the total WaLIDD score decreased by 62.4% at Cycle 3, whereas the placebo group showed progressive worsening, with a 17.1% increase from baseline. Across all WaLIDD subdomains, the test group demonstrated consistent and statistically significant improvements (p < 0.0001), whereas the placebo group showed no improvement or progressive deterioration. Across the clinical and observational literature evaluating ginger for primary dysmenorrhea, pain and symptom outcomes have predominantly been assessed using tools such as the VAS, numerical rating scales, Likert scales, categorical symptom grading, or the Verbal Multidimensional Scoring (VMS) system rather than the WaLIDD scale [9,21,23,24]. To the best of our knowledge, the present study is among the first to incorporate the WaLIDD scale to evaluate the effects of ginger supplementation in women with primary dysmenorrhea. Unlike the VMS, which provides a global categorical grade based on working ability, systemic symptoms, and the need for analgesics, the WaLIDD scale provides a numeric, multidimensional score that integrates working ability, pain location, pain intensity, and days of pain, thereby enabling a more granular assessment of dysmenorrhea severity and treatment response. Validation studies have demonstrated that the WaLIDD scale has strong discriminatory and predictive ability for functionally significant dysmenorrhea, including outcomes such as the need for medical leave [16]. These findings suggest that the WaLIDD scale may offer advantages over the VMS for detecting clinically meaningful changes over time. In this context, the consistent improvement in WaLIDD scores observed in the present study provides additional support for the robustness and clinical relevance of the VAS-based reductions in pain.

In addition to pain relief, the intervention resulted in consistent improvements in menstrual symptom burden, as assessed using the MEDI-Q. The test group demonstrated a near-complete reduction in symptoms as early as Cycle 1, with a 93.8% reduction in the total MEDI-Q score, which was sustained through Cycle 3, with a 95.3% reduction. In contrast, the placebo group showed a delayed and less pronounced improvement, with reductions of 30.8%, 84.1%, and 89.2% across Cycles 1, 2, and 3, respectively. Although both groups had low absolute scores by the end of the study, the between-group differences remained statistically significant, indicating an earlier onset and greater magnitude of improvement in the ginger group. These findings are consistent with earlier clinical trials in which ginger significantly alleviated pain associated with primary dysmenorrhea and improved menstrual-related symptoms such as nausea, fatigue, and general discomfort, although most previous studies primarily relied on pain-intensity scales and simple symptom checklists rather than a comprehensive measure of menstrual distress [9,20,23]. By employing the MEDI-Q, the present study extends the existing evidence base by demonstrating that the benefits of ginger may extend beyond pain reduction to encompass a broader spectrum of menstrual symptoms, including both physical and emotional dimensions of distress. To the best of our knowledge, this is among the first studies to evaluate the effects of ginger on menstrual distress using a validated, multidimensional instrument specifically designed to comprehensively assess menstrual symptom burden. This approach provides a more nuanced understanding of the potential effects of ginger on overall menstrual health.

The reduction in menstrual fluid flow observed with ginger supplementation in the present study (31.9% reduction from baseline) was consistent with previous reports [21,25], which also demonstrated significant reductions in menstrual blood loss. However, unlike earlier studies that assessed menstrual blood loss using the Pictorial Blood Assessment Chart (PBAC), the present study used menstrual cups to obtain a quantitative estimate of menstrual fluid volume.

The exploratory biomarker analysis demonstrated a progressive reduction in PGF2α levels in the test group, with an 11.6% reduction at Cycle 3, in contrast to an increasing trend in the placebo group, which showed a 7.0% increase. Given the established role of PGF2α in uterine contractions and dysmenorrhea-associated pain [4,26], these findings provide a plausible mechanistic basis for the observed clinical benefits. Although previous literature has proposed inhibition of prostaglandin synthesis as a potential mechanism underlying the effects of ginger, direct clinical evidence evaluating circulating PGF2α levels in women with primary dysmenorrhea remains limited [9,12,20]. Simarmata et al. reported that red ginger extract reduced PGF2α concentrations from baseline and was associated with an improvement in menstrual pain severity in women with primary dysmenorrhea [27]. However, these findings should be interpreted cautiously because the study lacked a placebo or active control group, used an insufficiently characterized ginger preparation, and had a short intervention period limited to a single menstrual cycle. Additionally, estradiol levels remained stable in both groups throughout the study, suggesting that ginger supplementation did not appear to produce substantial changes in circulating estradiol levels under the conditions of the present study. This finding may be relevant when considering the potential advantages of a non-hormonal intervention compared with hormonal pharmacological treatments.

The standardized ginger extract demonstrated a favorable tolerability profile throughout the 90-day intervention period, with no clinically significant gastrointestinal adverse events reported. Symptoms commonly associated with ginger supplementation, including gastric discomfort, heartburn, and epigastric irritation, were not observed. No treatment-related discontinuations occurred, and the overall safety findings were further supported by the absence of clinically meaningful changes in laboratory parameters, vital signs, or urinalysis findings. Although the present study was not specifically designed to investigate formulation-dependent gastrointestinal outcomes, the observed tolerability may be partially attributable to the granulated delivery system used in the formulation. The granulated formulation may facilitate a more gradual release of ginger bioactives and thereby potentially reduce localized gastrointestinal mucosal exposure; however, this proposed mechanism would require confirmation in dedicated formulation or pharmacokinetic studies. The granulated delivery system also supported a twice-daily dosing schedule, which is simpler than the more frequent dosing schedules (every 6-8 hours) used in some previous studies [19-22] and may potentially facilitate treatment adherence.

Certain limitations should be acknowledged. The relatively small sample size may have affected the precision and generalizability of the findings, although the distribution of participants between the treatment groups was comparable. The reliance on self-reported outcomes introduces the potential for reporting bias; however, this limitation was mitigated by the use of validated assessment instruments and a double-blind study design. Although the study duration was sufficient to demonstrate efficacy across three consecutive post-dosing menstrual cycles, it may not have been adequate to fully assess long-term efficacy and safety outcomes. Additionally, the exploratory biomarker analyses, although informative, should be interpreted cautiously because of potential biological variability and limited statistical power to draw definitive mechanistic conclusions. The findings are most applicable to otherwise healthy women aged 20-30 years with primary dysmenorrhea; therefore, extrapolation to other age groups or populations should be undertaken with caution.

The present findings have potentially important clinical implications. The standardized ginger extract may represent a safe, non-hormonal, and well-tolerated option for women with primary dysmenorrhea, including those who do not respond adequately to or prefer to avoid NSAIDs or hormonal contraceptives. The multidimensional benefits observed, including reductions in pain severity, menstrual symptom burden, and menstrual fluid flow, support its potential utility as a broader management option for primary dysmenorrhea. The observed association between symptom improvement and reduced PGF2α levels provides additional mechanistic support for the potential role of ginger in dysmenorrhea management, although further adequately powered studies are warranted to establish this mechanistic relationship.

## Conclusions

In this randomized, double-blind, placebo-controlled clinical study, the standardized ginger extract demonstrated statistically significant and clinically meaningful improvements in outcomes associated with primary dysmenorrhea. The intervention resulted in consistent reductions in pain severity, dysmenorrhea severity, menstrual symptom burden, and menstrual fluid flow across multiple menstrual cycles. These clinical benefits were accompanied by reductions in PGF2α levels, providing supportive evidence for a potential biological mechanism of action, while estradiol levels remained unchanged, suggesting no substantial effect on circulating estradiol levels under the conditions of the present study. The ginger extract was well tolerated, with no clinically significant treatment-related adverse effects observed. Overall, these findings suggest that the investigated standardized ginger extract may represent a safe, well-tolerated, and potentially effective non-hormonal option for the management of primary dysmenorrhea.
